# Simple and Efficient Synthesis of Oligoetherdiamines: Hardeners of Epoxyurethane Oligomers for Obtaining Coatings with Shape Memory Effect

**DOI:** 10.3390/polym15112450

**Published:** 2023-05-25

**Authors:** Daria Slobodinyuk, Alexey Slobodinyuk, Vladimir Strelnikov, Dmitriy Kiselkov

**Affiliations:** 1 Institute of Technical Chemistry Ural Branch of the Russian Academy of Sciences, Academic Korolev 3, 614130 Perm, Russia; selivanovadg@gmail.com (D.S.); svn@itcras.ru (V.S.); dkiselkov@yandex.ru (D.K.); 2 Department of Chemical Engineering, Perm National Research Polytechnic University, Komsomolsky Prospekt, 29, 614990 Perm, Russia

**Keywords:** self-healing coating, shape memory polymer, urethane, epoxyurethane oligomer, amino termination oligomer

## Abstract

In this work, new polymers with a shape memory effect for self-healing coatings based on oligomers with terminal epoxy groups, synthesized from oligotetramethylene oxide dioles of various molecular weights, were developed. For this purpose, a simple and efficient method for the synthesis of oligoetherdiamines with a high yield of the product, close to 94%, was developed. Oligodiol was treated with acrylic acid in the presence of a catalyst, followed by the reaction of the reaction product with aminoethylpiperazine. This synthetic route can easily be upscaled. The resulting products can be used as hardeners for oligomers with terminal epoxy groups synthesized from cyclic and cycloaliphatic diisocyanates. The effect of the molecular weight of newly synthesized diamines on the thermal and mechanical properties of urethane-containing polymers has been studied. Elastomers synthesized from isophorone diisocyanate showed excellent shape fixity and shape recovery ratios of >95% and >94%, respectively.

## 1. Introduction

Corrosion of metals has long been a serious problem for industries around the world. The global cost of corrosion is USD 2.5 trillion—3.4% of the global Gross Domestic Product [1]. In China, the annual damage caused by corrosion is over USD 390 billion, which is equivalent to 4.2% of the Gross Domestic Product [1]. Corrosion costs can be reduced by up to 15% to 35% by corrosion prevention measures [2].

As a rule, one of the main corrosion prevention methods is applying a protective organic polymer coating on a metal surface. However, most synthetic polymers are susceptible to degradation and cross-linking when subjected to light and in an oxygen medium, i.e., in environmental conditions. In addition, a polymer coating can be destroyed by physical impact. In all these cases, the coating should be repaired properly; otherwise, the metal would be subjected to corrosion.

The repair of coatings by conventional methods, such as removal of the defective coating and recoating, is costly and labor-intensive. Therefore, there is a constant market demand for polymer materials for self-healing coating. Self-healing polymers are a class of materials that have the ability to repair micro-scale damage in the coating and restore the passive state of the metal substrate. In this case, the coating healing process needs very little human “assistance”, if any.

The promising technology of self-healing polymers provides new opportunities for coatings to restore their protective characteristics with minimal or no intervention [3,4]. Thus, shape memory polymers (SMPs) represent a new class of smart materials that can recover from temporary deformation to their original shape due to entropy processes facilitated by external stimuli such as heat [5,6]. Shape memory polymers return to their original shape after being altered by external stimulation, such as electric and magnetic fields, temperature, pH, and mechanical stress [5,6].

Shape memory polymers return to their original shape after being altered by external stimulation, such as electric and magnetic fields, temperature, pH, and mechanical stress [7,8,9,10,11,12,13,14,15,16,17].

Shape memory polymers have many applications in the aerospace industry [18], medicine [19,20,21], self-processing smart textiles [22,23], electronic devices [24], and structural assembly [25,26,27].

Recently, shape memory polymers (SMPs) have been used to produce self-healing coatings with corrosion protection. For example, there are several reports on the use of urethane-containing SMPs based on poly(ε-caprolactone) (PCL) as self-healing coatings on metal substrates [28,29,30]. These studies show that the shape memory effect can significantly narrow the crack, thereby reducing the area of metal in contact with aggressive media.

Polyurethanes are block copolymers consisting of alternating soft and hard segments, SS and HS, respectively [31,32]. These polymers are usually prepared in a two-step process. At the first stage, a prepolymer with isocyanate-terminal groups is synthesized by the interaction of oligodiol and diisocyanate, taken in a double excess. In the second stage, the prepolymer is cured with low molecular weight chain extenders to form polyurethanes (hardener–diol) or polyurethane ureas (hardener diamine). The structure of the soft segments is determined by oligodiols used for the synthesis of polyurethane foam [33]. The structure of the hard segments is determined by diisocyanates used in the synthesis of oligodiisocyanates and by low molecular weight chain extenders, diamines, or diols [34].

Recently, some approaches for the preparation of self-healing PUs have been developed. This research trend can be illustrated by the following examples: encapsulation [35,36,37,38,39], healing in the polymer bulk via dynamic interactions, including non-covalent π–π bonding [40,41,42], ionic interactions [43], and thermoreversible covalent bonds [44,45,46,47,48,49,50,51,52,53]. Moreover, the shape memory effect caused by the thermal phase transition of the polymer is successfully used in the design of polyurethane materials.

In [54], the preparation of shape memory polyurethane was reported. In this synthetic route, a crystallizing polyester, polycaprolactone diol, was used as the main initial component. The resulting polymer was considered to be suitable for self-healing polymer coating.

Thus, Gonzalez-Garcia et al. developed a self-healing polymer coating film based on polycaprolactone diol (PCL) [55]. In case the coating is damaged, the relaxation of the soft polymer phase takes place. This process triggers temperature-induced self-healing, followed by the restoration of the barrier properties. This way, the damaged area is self-recoated.

A similar approach was used by Jorcin et al. [56]. In this study, a self-healing polyurethane coating with polycaprolactone soft segments was developed. However, a self-healing phenomenon was observed only at a hard segment content of 12%.

In [57,58,59], self-healing coatings with a shape memory effect were suggested.

Thus, the coatings described in [55,56,57,58,59] are characterized by the presence of a crystalline phase. It is a necessary condition for the preparation of this type of self-healing material. In addition, the presence of the crystalline phase contributes to an increase in the barrier properties of the coatings, i.e., their stability with respect to aggressive media [60].

It is noteworthy that for high-quality coatings, the adhesive properties of the polymer should be rather high. However, the strength characteristics of polyurethanes and polyurethane ureas depend on humidity, as the isocyanate group of oligodiisocyanates can react with moisture.

To solve this problem and to reduce the toxicity of isocyanate-terminated compounds, shielding the isocyanate groups of oligodiisocyanates with epoxy alcohol is the most efficient approach. For example, 2,3-epoxy-1-propanol can be used for this purpose [61]. In this case, the isocyanate groups of the oligodiisocyanate and the hydroxyl group of 2,3-epoxy-1-propanol react to give epoxyurethane oligomer (EUO). The deformation and strength properties of the elastomers, based on these oligomers, are only slightly dependent on the presence of moisture.

The elastomers based on epoxyurethane oligomers have good mechanical and dielectric properties. In addition, the adhesive properties of these elastomers are much higher than those of polyurethanes and polyurethane ureas. Thus, these polymers are widely used as adhesives, polymer matrices for casting low-modulus compounds for various purposes, and biomedical materials.

The structure of elastomers, based on epoxy urethane oligomers, consists of alternating soft and hard urethane hydroxyl segments. The microphase separation and the formation of separate phases, or domains, is due to the difference in the polarity of the structural units, soft and hard segments. Domains play the role of a reinforcing nanofiller or can be considered as the nodes of a physical network. This phenomenon is the reason for the high-strength characteristics of the developed materials. In the domains, hydrogen bonds play a decisive role in stabilizing the structure of the hard phase. In this case, the structure of hard segments can affect the morphology of the polymer in general.

To date, the crystalline elastomers, based on epoxyurethane oligomers, were prepared. Most of these elastomers were synthesized from polyesters [62,63]. However, these coatings are known to be unstable in water and, hence, they cannot be used for anti-corrosion treatment of metals. For these purposes, coatings based on polyethers, for example, oligotetramethylene oxide diols, can be useful.

In our previous studies, the attempts to obtain polyether-based elastomers were described [64,65,66]. A curing agent with terminal amino groups was synthesized via nucleophilic substitution of the hydroxy groups of oligotetramethylene oxide diols with amino groups. The authors assume that a material with improved stress-deformation and strength characteristics can be obtained. This can be realized by increasing the polarity of the hard segments, which is proportional to the degree of microphase separation of soft and hard segments in the polymer.

The present study aims to obtain shape memory urethane-containing polymers based on epoxyurethane oligomers synthesized from polyethers.

## 2. Materials and Methods

### 2.1. Materials and Synthesis

#### 2.1.1. Materials

Acrylic acid (Merck; Darmstadt, Germany), *N*-(2-aminoethyl)piperazine (AEP) (Merck; Darmstadt, Germany), 2,4-toluene diisocyanate (TDI) (BASF; Ludwigshafen, Germany), isophorone diisocyanate (IPDI) (Evonik Chemistry Ltd.; Essen, Germany), oligotetramethylene oxide diol with M_n_~1008 g·mol^−1^, M_n_~1400 g·mol^−1^, and M_n_~2000 g·mol^−1^ (OTMO-1000; OTMO-1400; OTMO-2000)(BASF; Ludwigshafen, Germany), glycidol (grade pure, 99.0%)(Research Institute of Polymer Materials; Perm, Russia), and dibutyltin dilaurate (grade pure, 99.8%) (BASF; Ludwigshafen, Germany) were used without purification.

#### 2.1.2. Synthesis of OTMO-diAEP

**Figure 1:**
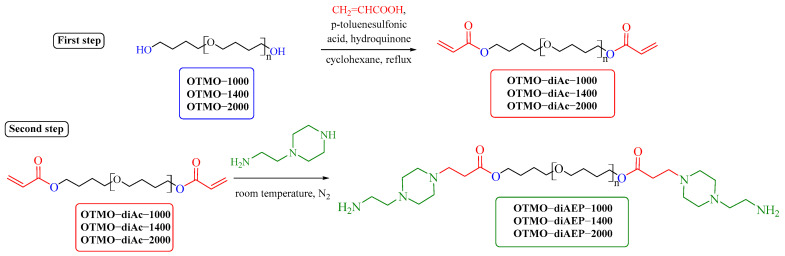
Synthesis of AEP functionalized OTMOs.

OTMO-diAEPs were prepared in two steps, shown in Figure 1.

In the first step, oligotetramethylene oxide diols (OTMO) were reacted with acrylic acid. Hydroquinone was added to prevent the copolymerization of acrylic acid with OTMO [67].

OTMO (0.02 mol), acrylic acid (0.052 mol), p-toluenesulfonic acid (0.002 mol), hydroquinone (0.0006 mol), and cyclohexane (320 mL) were placed in a round bottom flask with a Dean–Stark trap.

The reaction time was determined by the amount of water condensed in the Dean–Stark trap. The catalyst and acrylic acid residue was removed by adding potassium carbonate to the reaction mixture. The resulting mass was stirred at room temperature for 3 h. Then, the solution was filtered, and the solvent was distilled off on a rotary evaporator. The final products, OTMO diacrylates, were obtained in high yields (94%).

The second step of OTMO-diAEP preparation technique is the conjugated addition reaction of a cycloaliphatic diamine, aminoethylpiperazine, to a Michael acceptor, acrylate-terminated oligotetramethylen oxides. The addition reaction via the secondary amino group of aminoethylpiperazine is explained by the higher nucleophilicity of the secondary amino group compared to the primary [68].

OTMO-diAc (0.014 mol) was placed into a round bottom flask equipped with a magnetic stirrer. Further, aminoethylpiperazine (0.028 mol) was added to it. The reaction was carried out in a round-bottom flask equipped with a magnetic stirrer. The reaction mass was stirred in nitrogen medium for 2 h at room temperature. The product was isolated without purification. The product yield was 100%.

#### 2.1.3. Preparation of Epoxyurethane Oligomers 

**Figure 2:**
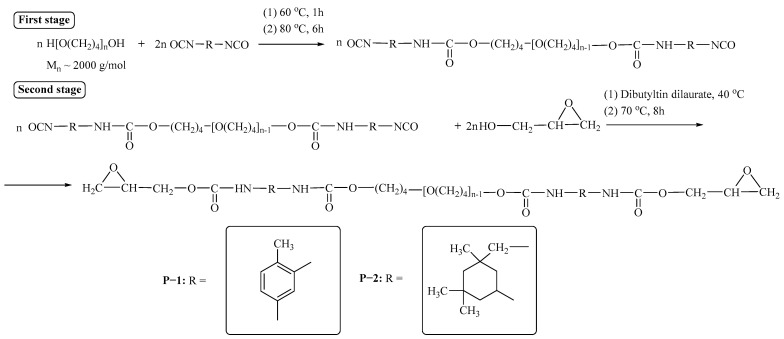
Preparation of P−1 and P−2.

The synthesis of epoxyurethane oligomers P−1 and P−2 was carried out in two steps (Figure 2).

OTMO was dried from water with constant stirring for 8 h at a temperature of 88–92 °C and absolute pressure in the reactor of 0.2–0.4 kPa. In the first stage, an oligomer with terminal epoxy groups was obtained by reacting the dried diol and diisocyanate, taken in a double excess at a temperature of 80 °C. The weight fraction of free isocyanate groups was determined according to ASTM D 2572-97. The second stage consisted of the treatment of the resulting oligomer with epoxy alcohol–glycidol in the presence of 0.03% urethane formation catalyst-di-n-butyl tin dilaurate. The reaction was carried out at a temperature of 70 ± 1 °C. Completion of the synthesis was determined by FTIR spectroscopy by the absence of an absorption band at 2270 cm^−1^ characteristic of the isocyanate group [69]. The mass fraction of free epoxy groups in the resulting oligomers was determined by the method described elsewhere [70].

The characteristics of the synthesized oligomers with various terminal groups are presented in Table 1.

#### 2.1.4. Preparation of Polymers

**Figure 3:**
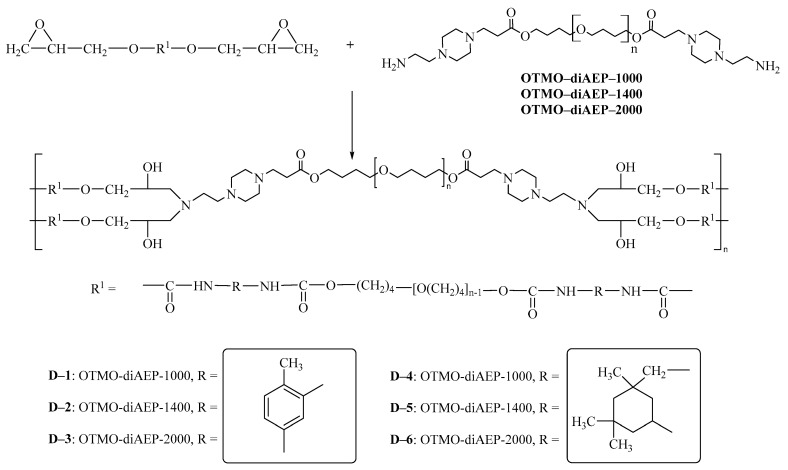
Synthetic route of polymers D−1–D−6.

Oligomers with terminal epoxy groups synthesized by a two-step method were cured with the developed diamines. To do this, the oligomer was stirred with diamine at a temperature of 50 °C for 5 min. The resulting mixture was thermostated at a temperature of 90 °C for 12 h. The disappearance of the absorption band at 910 cm^−1^ indicated the completeness of the transformation of the epoxy group [71]. The synthetic route is shown in Figure 3.

The polymer compositions are given in Table 2.

### 2.2. Methods

#### 2.2.1. ^1^H- and ^13^C-NMR Spectra

^1^H-NMR and ^13^C-NMR spectra were registered on a Bruker Avance Neo III spectrometer (^1^H: 400 MHz, ^13^C: 75 MHz) using tetramethyl silane as an internal standard. The chemical shift was calibrated with respect to the deuterium signal of CDCl_3_ at 7.26 ppm for ^1^H-NMR and 77.16 ppm for ^13^C-NMR.

#### 2.2.2. Elemental Analysis

Elemental analysis of CHN was performed on a Vario EL cube analyzer.

#### 2.2.3. Gel Permeation Chromatography (GPC)

The number average molecular weight of the oligomers obtained was measured using ULTIMATE 3000 HPLC chromatograph (Dionix Thermo Scientific, Moscow, Russia) equipped with a RefractoMax 521 refractometric detector, according to [72].

#### 2.2.4. FTIR Spectra

FTIR spectra were recorded using Vertex 80v spectrometer (Bruker, Moscow, Russia) equipped with A225/Q Platinum ATR unit at a 6 mm aperture, with a spectral resolution of 1 cm^−1^. The spectra were normalized using the band at 2860 cm^−1^, corresponding to symmetric vibrations of aliphatic–CH_2_ groups [73].

#### 2.2.5. Differential Scanning Calorimetry (DSC)

Heat effects in the samples within the temperature range from −100 °C to +100 °C were measured using a Mettler Toledo DSC 3+ calorimeter. Heating and cooling rates were 5 K min^−1^.

#### 2.2.6. Mechanical Tests

Mechanical tests of samples of the obtained materials were carried out on an Instron 3365 testing machine at the extension velocity υ = 0.417 s^−1^ and a temperature of 25 ± 1 °C according to the standard procedure. Based on the results of mechanical testing, the following characteristics were determined: σ_k_ (MPa)—the nominal strength (the maximal stress per initial specimen cross section); ε_k_ (%)—the relative critical strain; E_100_ (stress at the relative strain ε = 100%)—the nominal elastic modulus; and f_r_—the true tensile strength (f_r_ = σ_k_⋅λ_k_, where λ_k_ = (ε_k_ + 100)/100). The polymer was subjected to 5 tests.

#### 2.2.7. Shape Memory Properties 

The shape memory properties were measured by the bending test. The test was conducted in four steps, as described below. First, the sample was deformed to the U shape in a 50 °C lab oven. Then, the U shape sample was fixed in a freezing chamber at −10 °C for 5 min. After that, the sample was placed in a refrigerator at 0 °C for 5 min, and the angle after fixing (θ*_A_*) was measured. Finally, the sample was heated to 50 °C in the lab oven, and the angle after recovery (θ*_B_*) was measured. Before measuring θ*_A_* and θ*_B_*, the sample was held for 1 min at room temperature (25 °C). The shape fixing ratio and the shape recovery ratio are defined from θ*_A_* and θ*_B_*, respectively, as shown below:

$$\mathrm{Shape}\;\mathrm{fixity}\;\mathrm{ratio}\;(\%)=\frac{\theta_{A}}{180}\cdot 100$$

$$\mathrm{Shape}\;\mathrm{recovery}\;\mathrm{ratio}\;(\%)=\frac{180-\theta_{B}}{180}\cdot 100$$

## 3. Results and Discussion

### 3.1. NMR Measurement of OTMO, OTMO-diAc, and OTMO-diAEP

**Figure 4:**
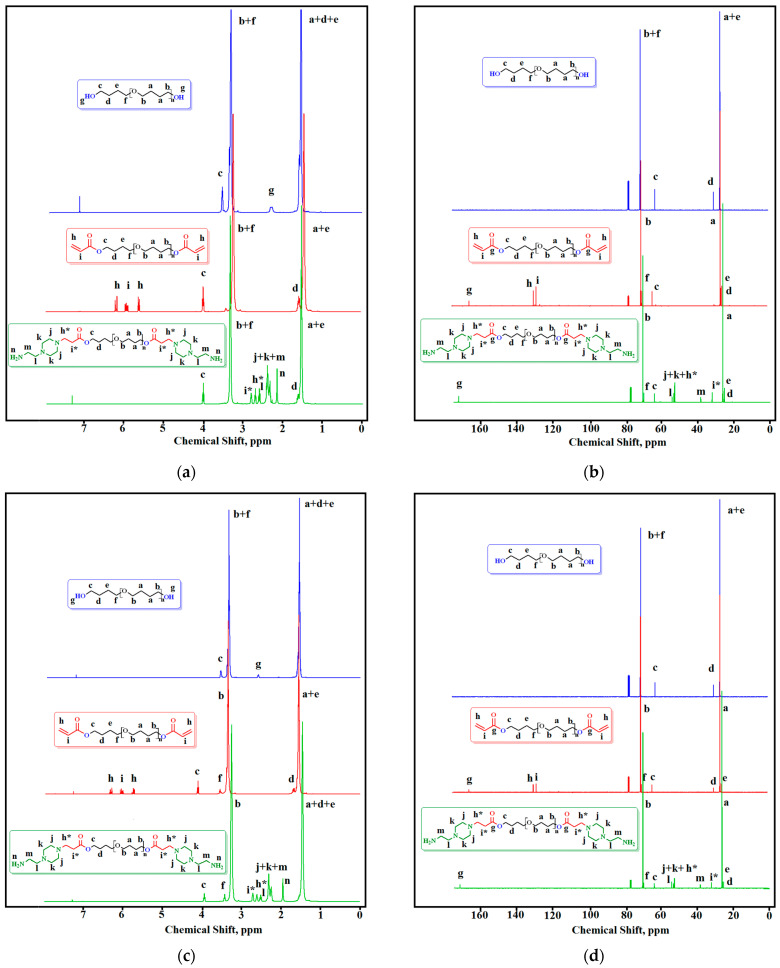

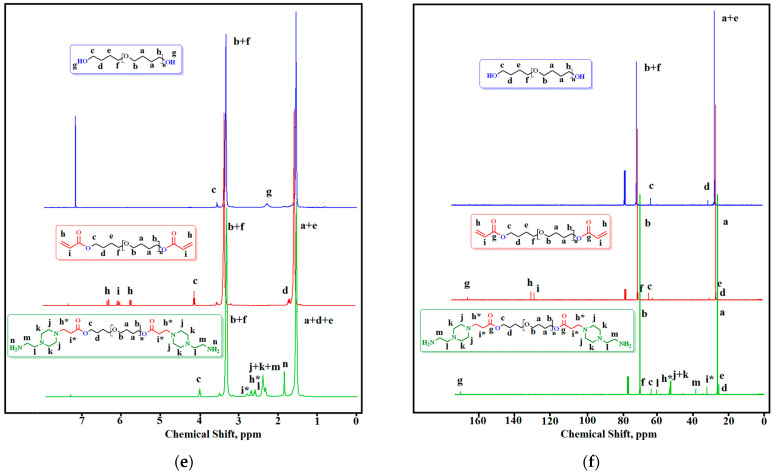
Nuclear magnetic resonance spectra (^1^H-NMR and ^13^C-NMR): (**a**,**b**) OTMO-1000, OTMO-diAc-1000, and OTMO-diAEP−1000; (**c**,**d**) OTMO-1400, OTMO-diAc-1400, and OTMO-diAEP−1400; (**e**,**f**) OTMO-2000, OTMO-diAc-2000, and OTMO-diAEP−2000.

The ^1^H and ^13^C NMR spectra of OTMOs, OTMO-diAcs, and OTMO-diAEPs are presented in Figure 4 and in Table S1 (Supplementary Materials).

In the ^1^H-NMR spectra, the signals of the methylene protons of oligotetramethylene oxide diols are at 1.51–1.62 (a + d + e) and 3.29–3.38 (b + f)) ppm. The protons of the hydroxyl groups are observed at 2.31 ppm (OTMO-1000), 2.60 ppm (OTMO-1400), and 2.29 ppm (OTMO-2000). The hydroxyl group signals disappeared upon the acrylation of the oligotetramethyleneoxide diols, and vinyl protons of terminal acrylate groups (h + i) appeared in the region of 5.75–6.35 ppm. The shift is caused by the change in the chemical environment of these protons, as the terminal hydroxyls of the oligomer chain were converted into acrylate groups. Upon the further reaction of OTMO-diAcs with aminoethylpiperazine, no vinyl protons of terminal acrylate groups were detectable. As a consequence, the triplets at 2.60 and 2.70 ppm (h* and i*) appeared. In addition, the signals of amino group protons could be observed at 2.16, 1.94, and 1.92 ppm (n). Both the multiplet at 2.23–2.43 ppm and the signal at 2.50 ppm were assigned to the protons of the aminoethylpiperazine fragment (j + k + m + l).

In the ^13^C-NMR spectra, there is a shift in the signals of terminal methylene carbons (c + d) during the sequential transformation of oligotetramethylene oxide diols into OTMO-diAEP. Additionally, for OTMO-diAc, the signals of the vinyl carbons of the acrylate groups appear in the downfield region (δ = 128 ppm (i), 130 ppm (h)). These signals shift upfield after the aza addition of the aminoethylpiperazine is completed (δ = 32 ppm (i*) and 53 (h*) ppm). In the ^13^C NMR spectra of OTMO-diAEP, the signals of the methylene carbons of aminoethylpiperazine fragment are observed at 38, 52, 53, and 61 ppm.

### 3.2. Elemental Analysis

The results of the elemental analysis are presented in Table 3. The experimental values turned out to be close to the theoretical values. The obtained data confirm the structure of all new compounds.

### 3.3. Gel Permeation Chromatography of OTMO-diAc and OTMO-diAEP

According to the results of gel permeation chromatography, the retention time of the synthesized compounds was determined, the values of which were from 5.04 to 6.58 min (Table 4). The obtained data indicate a narrow molecular weight distribution of compounds. Table 4 shows the average molecular weight of compounds determined by ^1^H-NMR spectroscopy and gel permeation chromatography.

### 3.4. FTIR Spectroscopy

#### 3.4.1. FTIR Spectroscopy of OTMO, OTMO-diAc, and OTMO-diAEP

**Figure 5:**
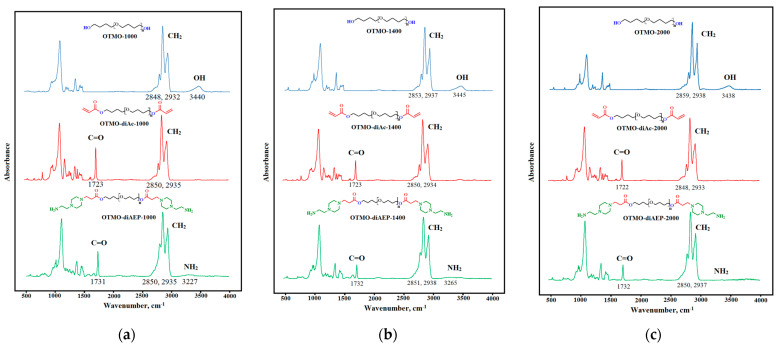
The Fourier-transform infrared spectroscopy spectra: (**a**) OTMO-1000, OTMO-diAc-1000, and OTMO-diAEP−1000; (**b**) OTMO-1400, OTMO-diAc-1400, and OTMO-diAEP−1400; and (**c**) OTMO-2000, OTMO-diAc-2000, and OTMO-diAEP−2000.

The FTIR spectra of OTMOs, OTMO-diAc, andOTMO-diAEPs are presented in Figure 5.

Acrylation of oligotetramethylene oxide diols leads to the appearance of a peak of carbonyl groups in the region of 1723 cm^−1^, while the absorption band of hydroxyl groups disappears. Further interaction of OTMO-diAc with aminoethylpiperazine leads to the appearance of an absorption band of amino groups, as well as to a slight shift in the peak of carbonyl groups. The remaining bands of intermediates and final products of OTMO-diAEP are identical to the bands of the starting oligotetramethylene oxide diols. According to the results of FTIR spectroscopy, it was proved that during the two-stage synthesis of OTMO-diAEP, only the end groups of oligomers changed.

#### 3.4.2. FTIR Spectroscopy of the Synthesized Elastomers

**Figure 6:**
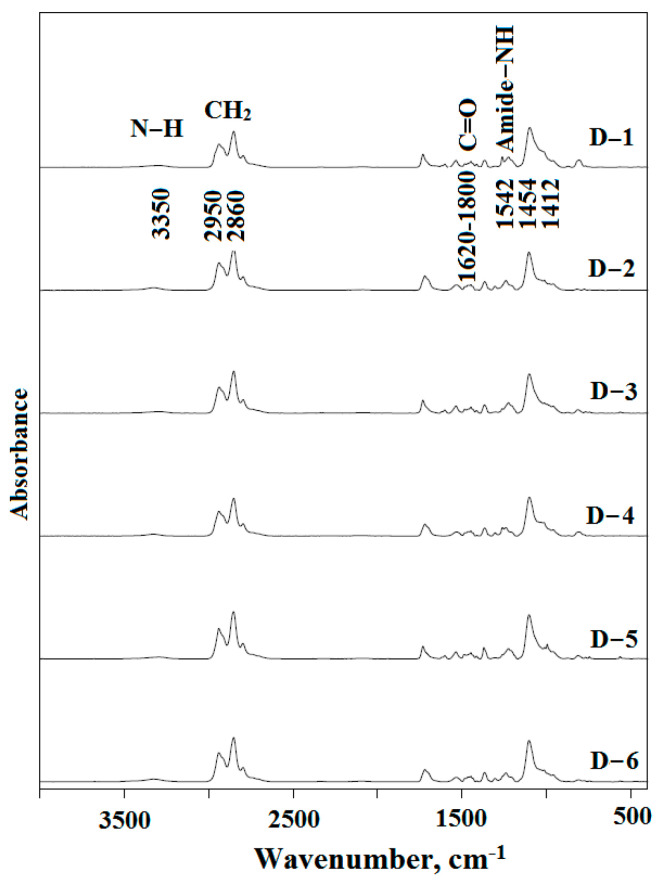
The Fourier-transform infrared spectroscopy spectra D−1, D−2, D−3, D−4, D−5, and D−6.

The overall spectrum of synthesized elastomers is shown in Figure 6. Elastomers synthesized from different diisocyanates are characterized by the same absorption bands: 3350 cm^−1^—NH band of urethane; 1542, 1454, 1412 cm^−1^—amide-NH stretching; 2860 cm^−1^—CH_2_ group; and also, 2950 cm^−1^—CH asymmetric stretching. However, there are also differences: for elastomers based on cyclic diisocyanate (2,4-toluylene diisocyanate), absorption bands at 1600 cm^−1^ and 1612 cm^−1^ appear in the spectrum. More detailed differences in the supramolecular structure of elastomers will be analyzed below when describing the FTIR spectra in the absorption region of carbonyl at 1600–1760 cm^−1^.

Revealing the features of the supramolecular structure of elastomers is possible by analyzing the FTIR spectra, namely, the region of carbonyl stretching vibrations (1600–1800 cm^−1^). It is known that the absorption band at 1695 cm^–1^ characterizes self-associates of rigid urethane hydroxyl blocks based on isophorone diisocyanate, and at 1705 cm^−1^ based on cyclic diisocyanate-2,4-toluene diisocyanate [64,74]. The intensity of these absorption bands determines the degree of microphase separation in elastomers.

**Figure 7:**
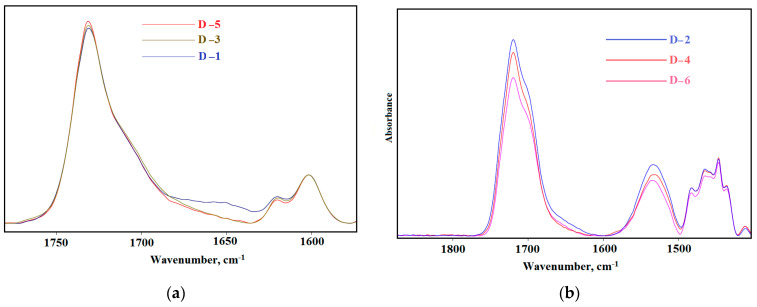
The Fourier-transform infrared spectroscopy spectra in the range of carbonyl stretching vibrations: (**a**) D−1, D−3, and D−5; (**b**) D−2, D−4, and D−6.

Figure 7 shows the analysis of IR spectra in the range of carbonyl stretching vibrations (1620–1800 cm^−1^). It is shown that elastomers based on isophorone diisocyanate have two pronounced absorption bands—at 1695 cm^−1^ and 1730 cm^−1^.

Elastomers based on 2,4-toluylene diisocyanate have one strong absorption band at 1730 cm^−1^ (Figure 7a). The absorption band at 1705 cm^−1^ appears weakly in the form of a shoulder. The lower intensity of this band indicates a lower degree of microphase separation of soft and hard segments in elastomers based on 2,4-toluylene diisocyanate.

### 3.5. Thermal Properties of the Synthesized Elastomers

**Figure 8:**
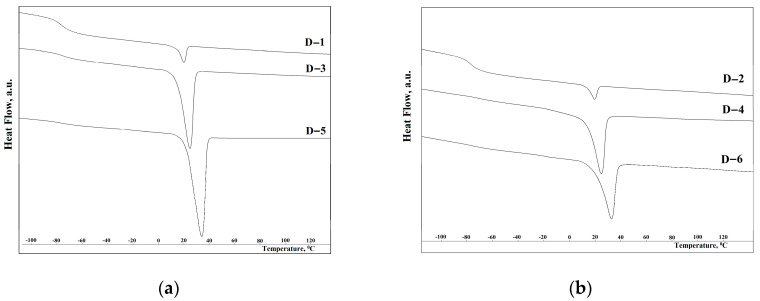
Differential scanning calorimetry data: (**a**) D−1, D−3, and D−5; (**b**) D−2, D−4, and D−6.

The DSC curves for elastomers D−1, D−2, D−3, D−4, D−5, and D−6 are shown in Figure 8. The thermal effects of elastomers were recorded according to the regime indicated in the work [64]: first, the samples were heated to 150 °C, then cooled to 100 °C below zero, kept for 30 min, and heated at a heating rate of 5 °C/min. 

The DSC results show that with an increase in the molecular weight of the hardener from 1374 to 1766 g/mol, the melting enthalpy of the elastomer increases by more than 10 times. A further increase in molecular weight does not lead to such a significant effect. It should be noted that higher melting enthalpies are realized on samples synthesized from 2,4-toluylene diisocyanate. This is due to the steric hindrance of the bulkier hard segment structure based on isophorone diisocyanate.

A higher degree of microphase separation of soft and hard segments of elastomers based on isophorone diisocyanate (Figure 8) makes it possible to obtain polymers with a lower glass transition temperature. At the same time, the value of the glass transition temperature less than minus 70 °C makes it possible to use these polymers under the extreme conditions of the Arctic and the Far North.

The derivative thermogravimetric curves of the elastomers D−1, D−2, D−3, D−4, D−5, and D−6 are shown in Figures S1–S6 (Supplementary Materials) and in Table 5.

The process of decomposition of samples, regardless of the molecular weight of the hardener, and the diisocyanate used in the synthesis of urethane-containing oligomers with terminal epoxy groups, occurs in two stages. The first stage occurs in the temperature range of 220–300 °C, and the second stage at temperatures of 320–360 °C, at a maximum weight loss temperature of about 310–320 °C.

### 3.6. Physical-Mechanical Characteristics of the Elastomers

According to the results of mechanical tests, it was proved that the molecular weight of the hardeners synthesized for the first time in this work affects the deformation properties of elastomers (Table 6). It has been established that elastomers synthesized on the basis of isophorone diisocyanate are characterized by the highest values of the relative critical strain.

It should be noted that with an increase in the molecular weight of the hardener from 1374 to 1766 g/mol, an increase in the nominal elastic modulus of elastomers is observed.

### 3.7. Shape Memory Properties of the Elastomers

The ratios of the shape memory fixity (performed at −10 °C) and the shape memory recovery (performed at 50 °C) measured at 25 °C (Figure S7 in Supplementary Materials) are listed in Table 7.

The results show that polymers cured with 1400 and 2000 molecular weight oligoamines have a high shape fixity ratio. At the same time, the Shape recovery ratio is higher for the samples synthesized from isophorone diisocyanate. The recovery process of all scaffolds at 50 °C was finished in 60 s.

### 3.8. Self-Healing Capability of the Prepared Coatings

**Figure 9:**
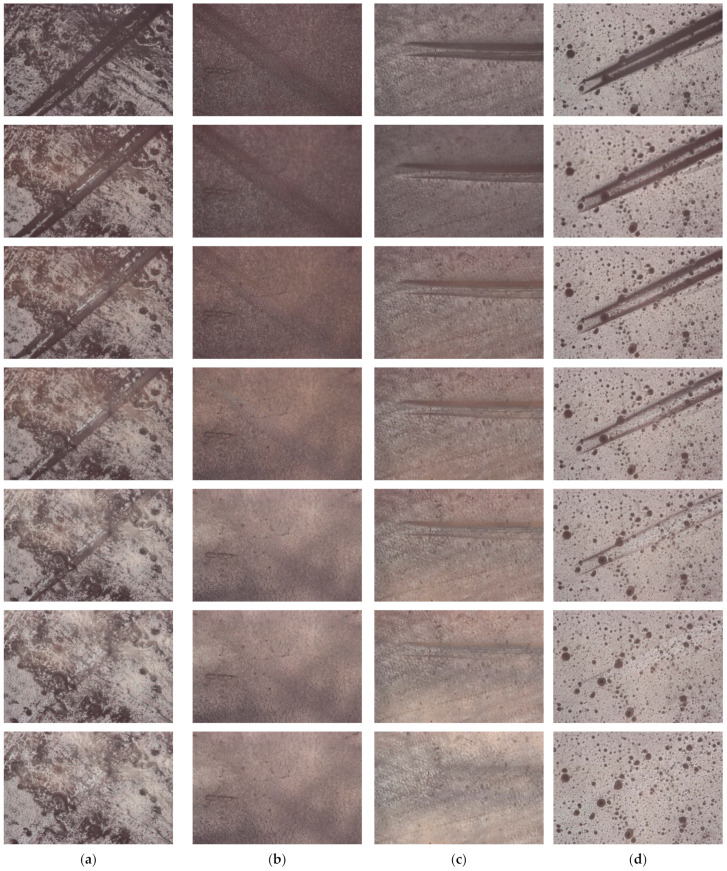
Optical micrographs for the samples: (**a**) D−3; (**b**) D−4; (**c**) D−5; and (**d**) D−6.

The self-healing capability of the prepared coatings was visualized using an optical microscope Olympus BX-51 (Figure 9).

The prepared coatings were scratched with a spatula (d = 1 mm). As a result, plastic deformation of the coatings occurred without the formation of cracks [75]. Next, the scratched coating was heated at a rate of 2 °C/min to 60 °C. As can be seen from Figure 9, with such heating, the plastic deformation is completely restored, and the scratch is healed.

Since no cracks formed on the coatings when using a spatula, the result obtained indicates the creation of materials with a reversible shape memory effect.

## 4. Conclusions

A simple and efficient method for the synthesis of amino-terminated oligotetramethylene oxides was developed, including the initial preparation of oligodiacrylates by reacting oligotetramethylene oxide diols with acrylic acid in the presence of a catalytic amount of p-toluenesulfonic acid and an inhibitor of acid polymerization, hydroquinone, and further reaction of the oligodiacrylate with a cycloaliphatic diamine by the conjugated addition method. The yield of target oligoetherdiamines was 94%.

Two epoxyurethane oligomers were prepared using the oligotetramethylene oxide diol with Mn~2000 g∙mol^−1^, isophorone diisocyanate, 2,4-toluenediisocyanate, and epoxy alcohol–glycidol.

Six elastomers from urethane-containing elastomers with terminal epoxy groups were prepared using synthesized amines.

The degree of microphase separation is higher for samples synthesized from isophorone diisocyanate.

It has been proven that the lower cleavage temperature of elastomers based on isophorone diisocyanate is realized due to a higher degree of microphase separation compared to elastomers based on 2,4-toluene diisocyanate.

At the same time, the glass transition temperature of elastomers less than 72 °C allows them to be used in the extreme conditions of the Far North.

The ability of the coating based on the developed polymers to self-healing has been demonstrated.

## Figures and Tables

**Table 1:** Characteristics of the epoxyurethane oligomers P−1 and P−2.

EUO Code	Molecular Weight of Initial OTMO	Diisocyanate	Content of Free NCO Groups, wt %		Content of Free Epoxy Groups, wt %	
			Experimental	Theoretical	Theoretical	Experimental
P−1	2000	2,4-toluene diisocyanate	3.57	3.51 ± 0.03	3.51	3.47 ± 0.03
P−2	2000	isophorone diisocyanate	3.43	3.42 ± 0.03	3.54	3.49 ± 0.03

**Table 2:** Compositions of polymers D−1, D−2, D−3, D−4, D−5, and D−6.

Polymer Code	EUO Code	Hardener
D−1	P−1	OTMO-diAEP−1000
D−2	P−2	OTMO-diAEP−1000
D−3	P−1	OTMO-diAEP−1400
D−4	P−2	OTMO-diAEP−1400
D−5	P−1	OTMO-diAEP−2000
D−6	P−2	OTMO-diAEP−2000

**Table 3:** Elemental analysis data of OTMO-diAc and OTMO-diAEP.

	C, %		H, %		N, %	
	Experimental	Theoretical	Experimental	Theoretical	Experimental	Theoretical
OTMO-diAc (M_n_ = 1116 g/mol)	65.82	65.60	10.45	10.40	-	-
OTMO-diAc (M_n_ = 1508 g/mol)	66.03	65.88	10.88	10.58	-	-
OTMO-diAc (M_n_ = 2108 g/mol)	66.24	66.10	10.86	10.74	-	-
OTMO-diAEP (M_n_ = 1374 g/mol)	63.92	63.76	10.65	10.63	6.32	6.12
OTMO-diAEP (M_n_ = 1766 g/mol)	64.52	64.39	10.86	10.76	4.92	4.76
OTMO-diAEP (M_n_ = 2366 g/mol)	65.05	64.97	10.95	10.87	3.81	3.55

**Table 4:** The number average molecular weight of compounds determined via ^1^H-NMR spectroscopy and gel permeation chromatography.

	M_n_^1^		M_n_^2^		M_n_^3^	
	GPC	^1^H-NMR **	GPC	^1^H-NMR **	GPC	^1^H-NMR **
OTMO-diAc	1104 (5.041 *)	1116	1479 (5.491 *)	1508	2079 (6.211 *)	2108
OTMO-diAEP	1391 (5.385 *)	1374	1747 (5.812 *)	1766	2387 (6.580 *)	2366

M_n_^1^—the number average molecular weight of OTMO-diAc-1000 and OTMO-diAEP−1000; M_n_^2^—the number average molecular weight of OTMO-diAc-1400 and OTMO-diAEP−1400; M_n_^3^—the number average molecular weight of OTMO-diAc-2000 and OTMO-diAEP−2000; *—retention time, min; and **—by ^1^H NMR, using the intensity of the signals of the terminal groups and repeating units.

**Table 5:** Thermal properties of the elastomers D−1, D−2, D−3, D−4, D−5, and D−6.

Composition Code	Glass Transition Temperature of the Soft Phase, °C	Melting Temperature of the Soft Phase, °C	Enthalpy of Melting ∆H_m_, J/g
D−1	−76	20	4.59
D−2	−76	18	3.41
D−3	−72	24	45.49
D−4	−74	24	40.88
D−5	−72	35	58.66
D−6	−74	35	48.63

**Table 6:** Deformation and strength characteristics of the elastomers D−1, D−2, D−3, D−4, D−5, and D−6.

Elastomer Code	σ_k_, MPa	ε_k_, %	E_100_, MPa	*f*_r_, MPa
D−1	10.20 ± 0.10	1150 ± 20	1.22 ± 0.05	127.5
D−2	11.50 ± 0.10	1023 ± 20	0.60 ± 0.05	129.2
D−3	9.10 ± 0.10	928 ± 20	2.28 ± 0.05	93.6
D−4	9.04 ± 0.10	914 ± 20	1.15 ± 0.05	91.7
D−5	8.96 ± 0.10	890 ± 20	3.28 ± 0.05	88.7
D−6	9.42 ± 0.10	830 ± 20	2.18 ± 0.05	87.6

**Table 7:** Shape fixity ratios and shape recovery ratios for elastomers.

Elastomer Code	Shape Fixity Ratio, %	Shape Recovery Ratio, %
D−3	95 ± 3	87 ± 3
D−4	96 ± 3	94 ± 3
D−5	95 ± 3	91 ± 3
D−6	97 ± 2	96 ± 3

## Data Availability

The most significant data generated or analyzed during this study are included in this published article. Further results obtained during the current study are available from the corresponding author upon reasonable request.

## Supplementary Materials

The following supporting information can be downloaded at https://www.mdpi.com/article/10.3390/polym15112450/s1. Figure S1: DTG curves D−1; Figure S2: DTG curves D−2; Figure S3: DTG curves D−3; Figure S4: DTG curves D−4; Figure S5: DTG curves D−5; Figure S6: DTG curves D−6; Figure S7: Shape Memory Properties of the elastomers D−3, D−4, D−5, and D−6; and Table S1: ^1^H-NMR and ^13^C-NMR data in CDCl_3_ for OTMO-diAc and OTMO-diAEP.
