# Supplementary material for: Simple and Efficient Synthesis of Oligoetherdiamines: Hardeners of Epoxyurethane Oligomers for Obtaining Coatings with Shape Memory Effect
Source: Polymers (Basel). 2023 May 25;15(11):2450. doi: 10.3390/polym15112450 (PMC10255675; doi:10.3390/polym15112450)
Supplement: Supplementary file 1 [file polymers-15-02450-s001.zip › polymers-2391163-supplementary.pdf]

Supplementary Materials

Simple and efficient synthesis of oligoetherdiamines: hardeners of epoxyurethane oligomers for obtaining coatings with shape memory effect

Daria Slobodinyuk [1], Alexey Slobodinyuk [1,2]*, Vladimir Strelnikov [1], Dmitriy Kiselkov [1]

[1] Institute of Technical Chemistry Ural Branch of the Russian Academy of Sciences, Ac. Korolev 3, 614130 Perm, Russia; svn@itcras.ru (V.S.); dkiselkov@yandex.ru (D.K.); selivanovadg@gmail.com (D.S.)
[2] Department of Chemical Engineering, Perm National Research Polytechnic University, Komsomolsky prospekt, 29, 614990 Perm, Russia
* Correspondence: slobodinyuk.aleksey.ktn@mail.ru (A.S.); Tel.: +7-(342)-2378256

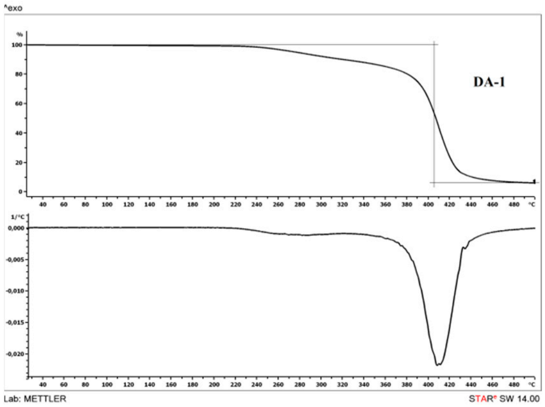

Figure S1. DTG curves D−1

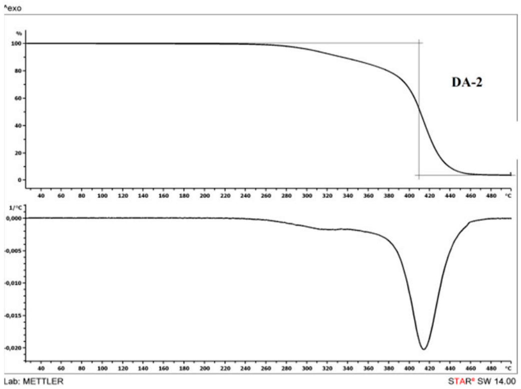

Figure S2. DTG curves D−2

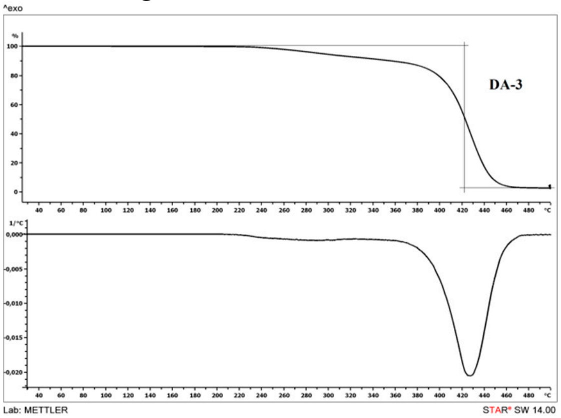

Figure S3. DTG curves D−3

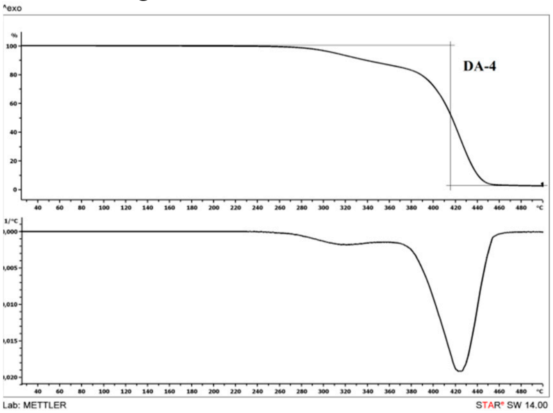

Figure S4. DTG curves D−4

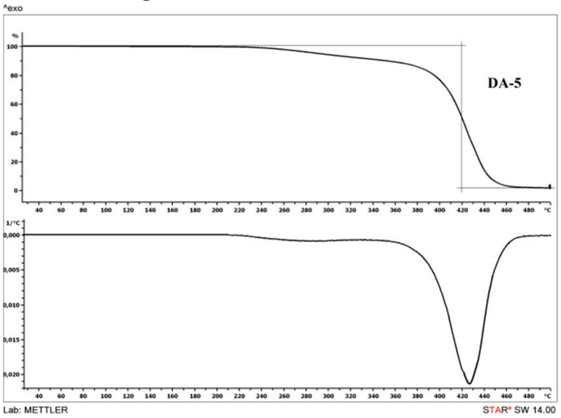

Figure S5. DTG curves D−5

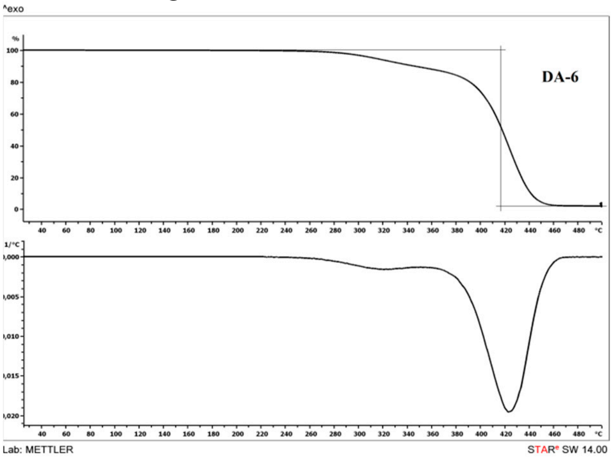

Figure S6. DTG curves D−6

Table S1. ^{1}H-NMR and ^{13}C-NMR data in $CDCl_3$ for OTMO−diAc and OTMO−diAEP.

Compound	^{1}H-NMR ($CDCl_3$, 400 MHz)	^{13}C-NMR ($CDCl_3$, 75 MHz)
**OTMO−diAc−1000**	1.44 (m; -O-CH$_2$-C*H*$_2$-C*H*$_2$-CH$_2$-O-, -O-CH$_2$-C*H*$_2$-CH$_2$-CH$_2$-O-C(O)-CH=CH$_2$; 56H), 1.57 (t; J 6.8 Hz; -O-CH$_2$-CH$_2$-C*H*$_2$-CH$_2$-O-C(O)-CH=CH$_2$; 4H), 3.23 (m; -O-C*H*$_2$-CH$_2$-CH$_2$-C*H*$_2$-O-, -O-C*H*$_2$-CH$_2$-CH$_2$-CH$_2$-O-C(O)-CH=CH$_2$; 56H), 3.99 (t; J 8.0 Hz; -O-CH$_2$-CH$_2$-CH$_2$-C*H*$_2$-O-C(O)-CH=CH$_2$; 4H), 5.62 (d; J 10.4 Hz; -O-C(O)-CH=C*H*$_2$; 2H), 5.90-5.97 (m; -O-C(O)-C*H*=CH$_2$; 2H), 6.19 (d; J 17.2 Hz; -O-C(O)-CH=C*H*$_2$; 2H)	25.2 (-O-CH$_2$-CH$_2$-*C*H$_2$-CH$_2$-O-C(O)-CH=CH$_2$), 25.8 (-O-CH$_2$-*C*H$_2$-CH$_2$-CH$_2$-O-C(O)-CH=CH$_2$), 26.1 (-O-CH$_2$-*C*H$_2$-*C*H$_2$-CH$_2$-O-), 63.8 (-O-CH$_2$-CH$_2$-CH$_2$-*C*H$_2$-O-C(O)-CH=CH$_2$), 69.6 (-O-*C*H$_2$-CH$_2$-CH$_2$-CH$_2$-O-C(O)-CH=CH$_2$), 70.1 (-O-*C*H$_2$-CH$_2$-CH$_2$-*C*H$_2$-O-), 128.2 (-O-C(O)-CH=*C*H$_2$), 129.8 (-O-C(O)-*C*H=CH$_2$), 165.5 (*C*=O)
**OTMO−diAc−1400**	1.55 (m; -O-CH$_2$-C*H*$_2$-C*H*$_2$-CH$_2$-O-, -O-CH$_2$-C*H*$_2$-CH$_2$-CH$_2$-O-C(O)-CH=CH$_2$; 80H), 1.69 (t; J 7.2 Hz; -O-CH$_2$-CH$_2$-C*H*$_2$-CH$_2$-O-C(O)-CH=CH$_2$; 4H), 3.35 (m; -O-C*H*$_2$-CH$_2$-CH$_2$-C*H*$_2$-O-; 76H), 3.56 (t; J 6.0 Hz, -O-C*H*$_2$-CH$_2$-CH$_2$-CH$_2$-O-C(O)-CH=CH$_2$; 4H), 4.11 (t; J 6.8 Hz, -O-CH$_2$-CH$_2$-CH$_2$-C*H*$_2$-O-C(O)-CH=CH$_2$; 4H), 5.73 (d; J 11.6 Hz, -O-C(O)-CH=C*H*$_2$; 2H), 6.01-6.08 (m; -O-C(O)-C*H*=CH$_2$; 2H), 6.32 (d; J 18.8 Hz, -O-C(O)-CH=C*H*$_2$; 2H)	25.4 (-O-CH$_2$-CH$_2$-*C*H$_2$-CH$_2$-O-C(O)-CH=CH$_2$), 26.1 (-O-CH$_2$-*C*H$_2$-CH$_2$-CH$_2$-O-C(O)-CH=CH$_2$), 26.3 (-O-CH$_2$-*C*H$_2$-*C*H$_2$-CH$_2$-O-), 64.1 (-O-CH$_2$-CH$_2$-CH$_2$-*C*H$_2$-O-C(O)-CH=CH$_2$), 69.9 (-O-*C*H$_2$-CH$_2$-CH$_2$-CH$_2$-O-C(O)-CH=CH$_2$), 70.3 (-O-*C*H$_2$-CH$_2$-CH$_2$-*C*H$_2$-O-), 128.4 (-O-C(O)-CH=*C*H$_2$), 130.1 (-O-C(O)-*C*H=CH$_2$), 165.9 (*C*=O)
**OTMO−diAc−2000**	1.55 (m; -O-CH$_2$-C*H*$_2$-C*H*$_2$-CH$_2$-O-, -O-CH$_2$-C*H*$_2$-CH$_2$-CH$_2$-O-C(O)-CH=CH$_2$; 112H), 1.69 (t; J 8.0 Hz; -O-CH$_2$-CH$_2$-C*H*$_2$-CH$_2$-O-C(O)-CH=CH$_2$; 4H), 3.34 (m; -O-C*H*$_2$-CH$_2$-CH$_2$-C*H*$_2$-O-; 108H), 3.53 (t; J 6.0 Hz; -O-C*H*$_2$-CH$_2$-CH$_2$-CH$_2$-O-C(O)-CH=CH$_2$; 4H), 4.11 (t; J 6.8 Hz; -O-CH$_2$-CH$_2$-CH$_2$-C*H*$_2$-O-C(O)-CH=CH$_2$; 4H), 5.73 (d; J 10.4 Hz; -O-C(O)-CH=C*H*$_2$; 2H), 6.01-6.08 (m; -O-C(O)-C*H*=CH$_2$; 2H), 6.31 (d; J 17.2 Hz; -O-C(O)-CH=C*H*$_2$; 2H)	25.3 (-O-CH$_2$-CH$_2$-*C*H$_2$-CH$_2$-O-C(O)-CH=CH$_2$), 26.0 (-O-CH$_2$-*C*H$_2$-CH$_2$-CH$_2$-O-C(O)-CH=CH$_2$), 26.2 (-O-CH$_2$-*C*H$_2$-*C*H$_2$-CH$_2$-O-), 64.0 (-O-CH$_2$-CH$_2$-CH$_2$-*C*H$_2$-O-C(O)-CH=CH$_2$), 69.8 (-O-*C*H$_2$-CH$_2$-CH$_2$-CH$_2$-O-C(O)-CH=CH$_2$), 70.2 (-O-*C*H$_2$-CH$_2$-CH$_2$-*C*H$_2$-O-), 128.3 (-O-C(O)-CH=*C*H$_2$), 130.0 (-O-C(O)-*C*H=CH$_2$), 165.7 (*C*=O)
**OTMO−diAEP−1000**	1.55 (m; -O-CH$_2$-C*H*$_2$-C*H*$_2$-CH$_2$-O-, -O-CH$_2$-C*H*$_2$-CH$_2$-CH$_2$-O-C(O)-CH$_2$-CH$_2$-AEP; 56H), 1.62 (t; J 6.8 Hz; -O-CH$_2$-CH$_2$-C*H*$_2$-CH$_2$-O-C(O)-CH$_2$-CH$_2$-AEP; 4H), 2.16 (s; -N*H*$_2$; 4H), 2.33-2.43 (m; -N-(C*H*$_2$-C*H*$_2$)$_2$-N-, -N-(CH$_2$-CH$_2$)$_2$-N-C*H*$_2$-CH$_2$-NH$_2$; 20H), 2.61 (t; J 7.6 Hz; -N-(CH$_2$-CH$_2$)$_2$-N-CH$_2$-C*H*$_2$-NH$_2$; 4H), 2.71 (t; J 6.0 Hz; -O-C(O)-CH$_2$-C*H*$_2$-AEP; 4H), 2.82 (t; J 6.0 Hz, -O-C(O)C*H*$_2$-CH$_2$-AEP; 4H), 3.34 (m; -O-C*H*$_2$-CH$_2$-CH$_2$-C*H*$_2$-O-, -O-C*H*$_2$-CH$_2$-CH$_2$-CH$_2$-O-C(O)-CH$_2$-CH$_2$-AEP; 56H), 4.03 (t; J 6.4 Hz, -O-CH$_2$-CH$_2$-CH$_2$-C*H*$_2$-O-C(O)-CH$_2$-CH$_2$-AEP; 4H)	25.2 (-O-CH$_2$-CH$_2$-*C*H$_2$-CH$_2$-O-C(O)-CH$_2$-CH$_2$-AEP), 25.9 (-O-CH$_2$-*C*H$_2$-CH$_2$-CH$_2$-O-C(O)-CH$_2$-CH$_2$-AEP), 26.2 (-O-CH$_2$-*C*H$_2$-*C*H$_2$-CH$_2$-O-), 32.0 (-O-C(O)-*C*H$_2$-CH$_2$-AEP), 38.3 (-N-(CH$_2$-CH$_2$)$_2$-N-CH$_2$-*C*H$_2$-NH$_2$), 52.6 (-N-(*C*H$_2$-CH$_2$)$_2$-N-CH$_2$-CH$_2$-NH$_2$), 52.8 (-N-(CH$_2$-*C*H$_2$)$_2$-N-CH$_2$-*C*H$_2$-NH$_2$), 53.2 (-O-C(O)-CH$_2$-*C*H$_2$-AEP), 60.4 (-N- (CH$_2$-CH$_2$)$_2$-N-*C*H$_2$-CH$_2$-NH$_2$), 63.8 (-O-CH$_2$-CH$_2$-CH$_2$-*C*H$_2$-O-C(O)-CH$_2$-CH$_2$-AEP), 69.8 (-O-*C*H$_2$-CH$_2$-CH$_2$-CH$_2$-O-C(O)-CH$_2$-CH$_2$-AEP), 70.2 (-O-*C*H$_2$-CH$_2$-CH$_2$-*C*H$_2$-O-), 172.0 (*C*=O).
**OTMO−diAEP−1400**	1.45 (m; -O-CH$_2$-C*H*$_2$-C*H*$_2$-CH$_2$-O-, -O-CH$_2$-C*H*$_2$-CH$_2$-CH$_2$-O-C(O)-CH$_2$-CH$_2$-AEP; 84H), 1.94 (s; -N*H*$_2$; 4H), 2.23-2.30 (m; -N-(C*H*$_2$-	25.3 (-O-CH$_2$-CH$_2$-*C*H$_2$-CH$_2$-O-C(O)-CH$_2$-CH$_2$-AEP), 25.9 (-O-CH$_2$-*C*H$_2$-CH$_2$-CH$_2$-O-C(O)-CH$_2$-CH$_2$-AEP),

	C*H*$_2$)$_2$-N-, -N-(CH$_2$-CH$_2$)$_2$-N-C*H*$_2$-CH$_2$-NH$_2$; 20H), 2.50 (t; J 7.2 Hz; -N-(CH$_2$-CH$_2$)$_2$-N-CH$_2$-C*H*$_2$-NH$_2$; 4H), 2.59 (t; J 6.0 Hz; -O-C(O)-CH$_2$-C*H*$_2$-AEP; 4H), 2.70 (t; J 6.0 Hz; -O-C(O)C*H*$_2$-CH$_2$-AEP; 4H), 3.24 (m; -O-C*H*$_2$-CH$_2$-CH$_2$-C*H*$_2$-O-; 76H), 3.41 (t; J 6.0 Hz; -O-C*H*$_2$-CH$_2$-CH$_2$-CH$_2$-O-C(O)-CH$_2$-CH$_2$-AEP; 4H), 3.93 (t; J 6.0 Hz; -O-CH$_2$-CH$_2$-CH$_2$-C*H*$_2$-O-C(O)-CH$_2$-CH$_2$-AEP; 4H)	26.2 (-O-CH$_2$-CH$_2$-CH$_2$-CH$_2$-O-), 32.1 (-O-C(O)-CH$_2$-CH$_2$-AEP), 38.4 (-N-(CH$_2$-CH$_2$)$_2$-N-CH$_2$-CH$_2$-NH$_2$), 52.6 (-N-(CH$_2$-CH$_2$)$_2$-N-CH$_2$-CH$_2$-NH$_2$), 52.8 (-N-(CH$_2$-CH$_2$)$_2$-N-CH$_2$-CH$_2$-NH$_2$), 53.2 (-O-C(O)-CH$_2$-CH$_2$-AEP), 60.6 (-N-(CH$_2$-CH$_2$)$_2$-N-CH$_2$-CH$_2$-NH$_2$), 63.8 (-O-CH$_2$-CH$_2$-CH$_2$-CH$_2$-O-C(O)-CH$_2$-CH$_2$-AEP), 69.7 (-O-CH$_2$-CH$_2$-CH$_2$-CH$_2$-O-C(O)-CH$_2$-CH$_2$-AEP), 70.2 (-O-CH$_2$-CH$_2$-CH$_2$-CH$_2$-O-), 171.8 (*C*=O)
**OTMO−diAEP−2000**	1.55 (m; -O-CH$_2$-C*H*$_2$-C*H*$_2$-CH$_2$-O-, -O-CH$_2$-C*H*$_2$-CH$_2$-CH$_2$-O-C(O)-CH$_2$-CH$_2$-AEP; 116H), 1.92 (s; -N*H*$_2$; 4H), 2.34-2.43 (m; -N-(C*H*$_2$-C*H*$_2$)$_2$-N-, -N-(CH$_2$-CH$_2$)$_2$-N-C*H*$_2$-CH$_2$-NH$_2$; 20H), 2.61 (t; J 7.2 Hz; -N-(CH$_2$-CH$_2$)$_2$-N-CH$_2$-C*H*$_2$-NH$_2$; 4H), 2.71 (t; J 6.0 Hz; -O-C(O)C*H*$_2$-CH$_2$-AEP; 4H), 2.82 (t; J 6.0 Hz; -O-C(O)-C*H*$_2$-CH$_2$-AEP; 4H), 3.34 (m; -O-C*H*$_2$-CH$_2$-CH$_2$-C*H*$_2$-O-; 108H), 3.52 (t; J 6.0 Hz; -O-C*H*$_2$-CH$_2$-CH$_2$-CH$_2$-O-C(O)-CH$_2$-CH$_2$-AEP; 4H), 4.03 (t; J 6.4 Hz; -O-CH$_2$-CH$_2$-CH$_2$-C*H*$_2$-O-C(O)-CH$_2$-CH$_2$-AEP; 4H)	25.3 (-O-CH$_2$-CH$_2$-CH$_2$-CH$_2$-O-C(O)-CH$_2$-CH$_2$-AEP), 25.9 (-O-CH$_2$-CH$_2$-CH$_2$-CH$_2$-O-C(O)-CH$_2$-CH$_2$-AEP), 26.2 (-O-CH$_2$-CH$_2$-CH$_2$-CH$_2$-O-), 32.1 (-O-C(O)-CH$_2$-CH$_2$-AEP), 38.5 (-N-(CH$_2$-CH$_2$)$_2$-N-CH$_2$-CH$_2$-NH$_2$), 52.6 (-N-(CH$_2$-CH$_2$)$_2$-N-CH$_2$-CH$_2$-NH$_2$), 52.9 (-N-(CH$_2$-CH$_2$)$_2$-N-CH$_2$-CH$_2$-NH$_2$), 53.2 (-O-C(O)-CH$_2$-CH$_2$-AEP), 60.7 (-N-(CH$_2$-CH$_2$)$_2$-N-CH$_2$-CH$_2$-NH$_2$), 63.9 (-O-CH$_2$-CH$_2$-CH$_2$-CH$_2$-O-C(O)-CH$_2$-CH$_2$-AEP), 69.8 (-O-CH$_2$-CH$_2$-CH$_2$-CH$_2$-O-C(O)-CH$_2$-CH$_2$-AEP), 70.2 (-O-CH$_2$-CH$_2$-CH$_2$-CH$_2$-O-), 172.0 (*C*=O)

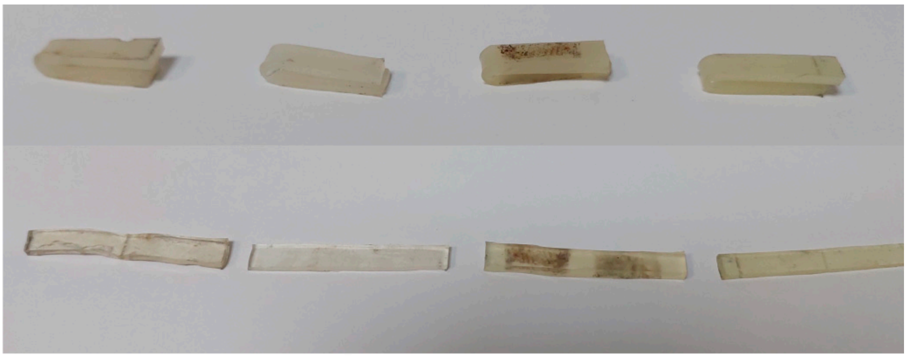

D-3 **D-4** **D-5** **D-6**

Figure S7. Shape Memory Properties of the elastomers D–3, D–4, D–5, D–6
